# Medical Heroism

**Published:** 1847-12

**Authors:** 


*Medical Heroism.*—Under this caption, the *Annalist* copies the following from an article in the *British and Foreign Review*, with appropriate comments. It is a sublime but just picture of genuine medical character.

"There are few of our readers who do not remember the melancholy impression made on the public mind by the disastrous expedition to the Niger, when this was made known in England, through the newspapers. And none, who remember this, can forget that pathetic passage in the story, which represented the noble conduct of the surgeon and the geologist of the expedition, when left alone in the far recesses of the Niger, amid their heroic companions, all stricken to death, or to death-like helplessness, by the fatal fever of the country. In this trying conjuncture, when the salvation of all on board depended upon the speedy removal of the ship from her actual position, Dr. M'William took the navigation on himself, steering with his own hands, and piloting the vessel through all the intricacies of the river, while his companion worked the engine below. There is something affecting, we had almost said, sublime, in the picture thus presented to the imagination, of these two solitary men of science, assuming offices so foreign to their past habits and knowledge, stripped of all exterior cognizance of their class, standing as humble workmen at the helm and furnace, toiling by day, watching by night, while the force of the stream and paddles were sweeping their ill-fated bark, freighted with their dying or dead companions, through the manifold dangers of their unknown course. The author of the volume before us, (the Report on the Boa Vista Fever,) was the clear-headed and stout-hearted pilot who did this, the undoubted preserver of the ship and her surviving crew; and the slight and simple way in which he speaks of his own exertions, strikingly illustrates the old truth, that 'the brave is ever modest.'"

The following dispatch and accompanying remarks, which we extract from the North American of a recet date, show that the same traits characterize the honorable members of our profession everywhere.

*Martyrs to their duty.*—The crowded state of our columns has prevented us from commenting upon the following dispatch received at the Navy Department from Com. Perry.

U. S. Flag Ship Germantown, Vera Cruz,
6th September, 1847.

Sir:—I am again called upon to announce to the Department the death of another valuable officer of the Squadron—Passed Assistant Surgeon J. Howard Smith breathed his last yesterday at the Naval Hospital.

The death of this and the other Medical Officers, may in part be ascribed to the extraordinary anxiety and labor to which they were subjected in their attendance upon the sick; worn out in body, though not in zeal and courage, they had not sufficient strength to bear up against the effects of disease when it came upon them.

Doctor Smith was attached to the steamer "Spitfire," and volunteered with Doctor Hastings, of the "Mississippi," to take charge of the sick at the Hospital, when Dr. Thornly was taken with the fever.

Words cannot express my feelings on seeing these devoted men, stricken down as they have been by the epidemic, from the fatal malignancy of which their own incessant labors and watching by night and by day have saved so many.

As a proof of the noble self-devotion of Dr. Hastings—an example worthy also the character of his lamented companion, Dr. Smith—I subjoin an extract from the "Sick Report" of the 30th ult.

I have the honor to be, with great respect, sir, your obedient servant, (Signed) M. C. Perry,
Commanding Home Squadron.

To Hon. John Y. Mason,
Secretary of the Navy, Washington.

Both the gentlemen who are the subject of the above feeling eulogy, are Philadelphians, and Mr. Smith leaves many to regret his loss—while that regret is solaced by the reflection that he fell when nobly and gallantly engaged in the performance of his duty. It is not alone amid the roar of cannon and the thick smoke of a deadly combat that true courage and manly devotedness can be found. The physician who faces the horrors of infectious disease, and hovers like a ministering angel around the couch of death, achieves a triumph equal to that of the victorious battle field. As soon as Dr. SMITH was taken down, Dr. JOHN HASTINGS made the following tender of his services in a report dated "U. S. Naval Hospital, Salamandina, August 30, 1847."

"Aware of the diminished number of Medical officers in the squadron, and fearing you might be worried and perplexed on account of the sickness of Dr. Smith, I conceive it my duty to say that I feel myself able to take charge of the sick at present on the island, (number of sick in hospital 124,) and all who will be likely to come. Having been on a previous occasion, from similar misfortunes, called upon to discharge as heavy and important a duty as the present without succumbing, I hope I shall in the present instance again be equal to the task.'

While engaged in the duties which he had thus assumed, Passed Assistant Surgeon JOHN HASTINGS was himself attacked by fever and for a long time his recovery was doubtful. Providence, however, interposed, and he has been preserved for other duties and other missions of self-sacrifice and devotion. Well may we feel proud to enrol such gallant spirits among the sons which Philadelphia has sent forth to the war. Tears for the departed one, and the encomiums of the public upon the survivor, are but feeble evidences of the estimation in which they are held. Dr. SMITH sleeps the sleep that knows no waking, while Dr. HASTINGS will soon be restored to the friends whom he has made so proud of him, and we trust his return may be accompanied by some expressive token from his fellow-citizens."

The following notice of the late Dr. Kearney we also take from the North American. We had the pleasure of Dr. K.'s acquaintance and friendship, during several years that he was stationed at Philadelphia, and can bear witness that the eulogy of his brother officers is not strained. It will be seen that he met his death in the *voluntary* performance of a most hazardous duty. Dr. Kearney's age and long service exempted him from so arduous a task, but his sympathy for his brother soldiers prevailed over all considerations of domestic ease and comfort, and even his own health and life, and, like those whose names we have already chronicled, he fell a sacrifice to what he deemed his professional duty.

NAVAL.—The Officers of the Navy attached to this station, and others now in Philadelphia, met at the Navy Yard, on the 22d inst. Commodore CHARLES STEWART was called to the Chair, and Purser ROBERT PETTIT appointed Secretary.

The Chairman, in a short and feeling address, stated that they had convened for the purpose of offering some testimonial of respect, to the memory of a brother officer, Doctor John A. Kearney, late "Fleet Surgeon" of the Home Squadron, who died on the 27th of last August, at Salamandina, in the Gulf of Mexico.

A Committee consisting of Surgeons James M. Greene, W. S. W. Ruschenberger, and Purser Robert Pettit, was appointed to prepare a notice and resolutions, expressive of the sentiments and feelings of the meeting. The Committee reported the following, which was adopted:

We are impressed with no common feelings of sorrow, when we reflect, that only a few short weeks have elapsed, since the lamented subject of this notice, was on duty with us, in fine health and spirits, and with every prospect of a long and useful life; and that now he is numbered among the last victims of that disease, the fell scourge of strangers within the tropics!

We cannot permit an occasion so melancholy to pass, without a slight tribute to the memory of one with whom most of us have passed many happy hours, in our varied duties of a life at sea; or amid the social circle, with our friends on shore.

Doctor Kearney entered the service at an early age, and during a career of nearly forty years, passed with distinguished honor through the various ranks of the Medical Corps of the Navy. He served as Surgeon's Mate, Surgeon, Member and President of Medical Boards, and Fleet Surgeon of different Squadrons.

In the war of 1812 he was Surgeon of the Frigate Constitution, in her triumphant encounter with two of the enemy's vessels and a larger force.

The deceased was a warm advocate for strict discipline, and willingly showed the utmost deference for those whose rank or seniority placed them in command—while he used every honorable means to promote the consideration which he believed was due to skilful and well instructed medical officers. Nor was he less strenuous in his efforts to advance the interests of others in the different departments of the service; and in 1835 had the pleasure to witness the passage of a law in Congress, improving the condition of every rank in the Navy.

Doctor Kearney was a noble hearted and generous man—he was a kind affectionate husband, a tender parent, most sincere friend, and acquitted himself in all the relations of life, as a christain and polished gentleman.

But it was in the exercise of his profession, either in the hospital, or on board his ship, that he was seen to most advantage—there, his whole time and attention were given to the sick, and no comfort or luxury was spared that could alleviate the sufferings of the patient, or tend to his recovery.

As friends of the deceased, we only join in the common expression of sorrow, at the loss which his family, the public service, and society have sustained in the death of Doctor Kearney—and it is therefore

*Resolved*, That we sympathise with his family and relatives in their bereavement, and that we offer them our heartfelt regret, and most sincere condolence.

*Resolved*, That when time shall have assuaged the sorrows of his mourning relatives, they may direct his children to contemplate with becoming pride, a father's patriotism, his unsullied reputation, and the bright example of his virtues.

*Resolved*, That we appreciate his services in co-operating with the Army, in its struggle with the ruthless Seminole of Florida, and in *voluntarily* joining the squadron, now amidst disease and death in the Gulf of Mexico.

*Resolved*, That we will cherish with grateful recollection the memory of the deceased, as a skilful and efficient officer, a pleasing associate on duty, and as an accomplished gentleman.

*Resolved*, That these proceedings be signed by the Chairman and Secretary, and published in the papers of this city, and that a copy of them be sent to the Secretary of the Navy, with a request that he forward it to the family of the deceased, and also that a copy of it be sent to the Secretary of War, for Col. James Kearney, of the Topographical Engineers, only surviving brother of the deceased.

Charles Stewart, Chairman.

*Robert Pettit*, Secretary.

U. S. Navy Yard, Philadelphia, Sept. 22d, 1847. *Med. Exam.*

